# Book Review

**Published:** 1931

**Authors:** 


### Reviews of Books

**Tuberculosis: its Treatment and Cure with the help of Umckaloabo.** By an English Physician. Pp. v., 153. London: B. Fraser & Co. 1931. Price 3s. 6d.—This records an investigation of the claims of Umckaloabo, manifestly with the co-operation of the proprietor, though we are not told at whose expense. The anonymous author "happens to be the medical adviser to a great London newspaper," nevertheless he displays such a positively childlike innocence in his account of the origin and properties of this mysterious root as proclaims him an ideal subject for "crural extension." Records are given of fifty-five cases, including forty-six of pulmonary tuberculosis. We are not told how they were selected, so it is fair to assume that they represent the best out of some 9,000. The cases are numbered, though on what system is obscure; No. 3,167 began treatment in 1911, 7,398 in 1912, 203, 1,904, 6,650 and 7,622 in 1913, 3,095 in 1919, 155 in 1921, 7,065 in 1922. There is no obvious order in their presentation, but close scrutiny brings to light the interesting fact that the first fifteen include twelve with "positive sputum," although the remaining forty show only nine "positives," and of these two had ceased to be positive before starting treatment. As regards the remainder, we are not impressed by such claims as "she had already been certified as having pulmonary tuberculosis, so there *can be no doubt whatever* of its existence." (Page 104.) Nearly every case had had sanatorium treatment with dispensary after-care, etc. In many, five, ten, even thirteen and fourteen years had elapsed between onset and commencement of Umckaloabo treatment. Each case was "examined" once only, often many years after the "cure" had been completed. The book is obviously intended to appeal to the ignorant and credulous; to the critical reader it is the most damning exposé of the futility of the "cure" it extols that has yet seen the light.

**Encephalitis Lethargica.** By Constantin von Economo. Pp. xvi., 200. 21 illustrations. London: Oxford University Press. 1931. Price 18s. This monograph represents the views of perhaps the greatest authority on the subject. It contains a complete historical survey, together with an exhaustive discussion of the present position of experimental work on the virus of encephalitis. As regards the latter no finality is reached. The classification of the clinical types of the disease is a distinct advance on that found in current English text-books. The author gives a detailed account of recent histological work on the lesions of encephalitis lethargica. He makes a great point of the influence of the vegetative centres in determining modes of thought and conduct and he sees, as the result of his researches, the birth of a new psychology. Great stress is laid on the influence of these vegetative centres, notably a sleep centre, but the argument is obscured by a complete failure to discriminate between positive and negative symptomatology. It is impossible to make out, from the writer's account, which sleep disturbances are due to overaction of the sleep regulating centre and which are due to its destruction—one cannot have it both ways. A similar vagueness pervades the author's remarks with regard to stimulation or destruction of other regions of the brain affected by encephalitis. However, apart from these defects, the book presents the most comprehensive account of the disease available at the present time.

**The Alcohol Habit and its Treatment.** By WALTER E. MASTERS, M.D. Pp. 190. London: H. K. Lewis & Co., Ltd. 1931. Price 6s. The purpose of Dr. W. E. Masters in this brochure is to recommend to the profession the treatment of alcoholism by drugs, in conjunction with a stay in a voluntary home reserved for that purpose. Such a method, he conclusively shows, does give a permanent satisfactory result in a high percentage of cases. The book is full of novel and interesting information, the chapters on the types of alcoholism and the motives and predisposing causes being especially valuable.

**The Medical Evidence before the Royal Commission on Licensing, 1930.** By COURTENAY C. WEEKS, M.R.C.S., L.R.C.P. Pp. x., 153. London: National Temperance League. 1931. Price 1s. Dr. Weeks's summary of the evidence given by medical witnesses before the Royal Commission on Licensing, 1930, should be read by all practitioners of medicine. Particularly the facts should be taught to medical students, for there is growing up a generation of medical men who are becoming increasingly careless and unscientific in the use of alcohol in the treatment of disease and as an ordinary article of diet. Although the summary is tinged with Dr. Weeks's well-known views on total abstinence, the question is fairly and frankly discussed. The chapters on the ill-effect of small doses of alcohol on all classes of skilled work, on alcohol in medical treatment and on its relation to child-life are particularly instructive. Dr. Salter's evidence on the social aspect of the question, founded on his experience in the Borough of Bermondsey, is a forcible reminder of the fact that the abuse of alcohol is still for a large section of the community a problem of vast dimensions and of utmost gravity.

**Restoration Exercises for Women.** By ETTIE A. HORNIBROOK. Pp. xi., 95. Illustrated. London: William Heinemann Ltd. 1931. Price 5s. This is an excellent but very expensive little book. It contains some dozen exercises which should be extremely useful to those troubled with constipation (and flat feet), if carried out diligently and in conjunction with the dietary given, which everyone might follow with advantage. It does not savour of crankiness as much as books of this type usually do. The diagrams are clear, the printing fair and there is a useful index, but the binding is shocking. The book is really a supplement to F. A. Hornibrook's *Culture of the Abdomen,* with which it might, with advantage, be re-issued in one volume at the same price.

**Notes on Radium Therapy for Medical Students.** By HECTOR A. COLWELL, M.B., Ph.D. Pp. xii., 165. Illustrated. London: H. K. Lewis & Co. Ltd. 1931. Price 6s. One can find but little fault with Colwell's *Notes on Radium Therapy.* The chapter on Physics is especially well written, and can be very easily understood by even the least mathematically inclined. It would be well, however, when this book is revised if on page 8 the more usual abbreviations of "kms" and "cms" are used instead of "kilos" and "cents." Although the writer states that the clinical chapters of the book are merely notes intended for final year students, they could be read with profit by all who want to know a fair amount about radium without actually wanting to specialize. The quotation of Birkett's figures in carcinoma of the tongue gives a rather misleading impression. These were not cures, but the percentage of cases clinically well at the end of a year from the time of treatment. As Colwell says elsewhere, there are no cures in the sense of five year figures available for England. It is a pity that except in the chapter on carcinoma of the cervix very little mention is made of techniques other than the English, while a few words on the "Bomb" treatment, a little more about surface treatment, and a passing reference to radium therapy in neoplasms of the stomach, scrotum, and brain would have still further enhanced the value of this excellent little book.

**Hay-fever, Hay-asthma.** By WILLIAM LLOYD, F.R.C.S. Third Edition. Pp. vii., 124. Illustrated. London: Straker Bros. Ltd. 1931. The first edition of this book appeared in 1907: the author has nothing startlingly new to say now, but what he does say is extremely valuable. He gives an adequate account of a very common and distressing complaint, its ætiology, and clinical and pathological manifestations. Emphasis is rightly laid on the importance of nasal treatment and on the equal importance of undertaking treatment early. The directions given are clear and adequate, and the case reports indicate how satisfactory is the prognosis. This book should certainly be in the library of every practitioner who does not know all about the cure of hay-fever.

